# Reviewing the Potential Therapeutic Approaches Targeting the Modulation of Gastrointestinal Microflora in Schizophrenia

**DOI:** 10.3390/ijms232416129

**Published:** 2022-12-17

**Authors:** Ilinca-Bianca Nita, Ovidiu-Dumitru Ilie, Alin Ciobica, Luminita-Diana Hritcu, Irina Dobrin, Bogdan Doroftei, Romeo Dobrin

**Affiliations:** 1 Department of Medicine III, Faculty of Medicine, University of Medicine and Pharmacy “Grigore T. Popa”, University Street, no 16, 700115 Iasi, Romania; 2 Department of Biology, Faculty of Biology, “Alexandru Ioan Cuza” University, Carol I Avenue, no 20A, 700505 Iasi, Romania; 3 Internal Medicine Clinic, Faculty of Veterinary Medicine, University of Life Sciences “Ion Ionescu de la Brad”, Mihail Sadoveanu Street, no 3, 700490 Iasi, Romania; 4 Institute of Psychiatry “Socola”, Bucium Street, no 36, 700282 Iasi, Romania; 5 Department of Medicine VIII, Faculty of Medicine, University of Medicine and Pharmacy “Grigore T. Popa”, University Street, no 16, 700115 Iasi, Romania

**Keywords:** schizophrenia, gut microflora, probiotics, prebiotics, synbiotics, fecal microbiota transplantation, microbial transfer therapy

## Abstract

Schizophrenia (SCZ) is a severe brain disorder characterized by an intriguing clinical panel that has begun to gain interest due to its particular phenotype. Having considered the role of gut microflora in psychiatry, the latest discoveries might offer further insight into the underlying mechanisms. Thus, we aimed to offer an updated overview of the therapeutic potential of microorganism-derived supplements alongside dedicated protocols that target the re-establishment of the host’s eubiosis. Based on combinations of specific keywords, we performed searches in four databases (PubMed/Medline, ISI Web of Knowledge, Scopus, and ScienceDirect) for the established interval (2018–2022) and identified twenty two eligible cases, restricted only to human patients’ experiences. Up until the writing of this manuscript, it has been revealed that the administration of specific lactic acid bacteria strains (*Lactobacillus* and *Bifidobacterium*), or those combined with vitamin D and selenium, maintain the integrity of the gut flora, preventing antagonistic effects including inflammation, antipsychotic-related body weight gain (olanzapine) and other metabolic dysfunctionalities. However, there are multiple antipsychotics that exert a potent effect upon gut flora, influencing a plethora of pathways and creating a dysbalance ratio between beneficial and opportunistic pathogens. Risperidone, amisulpride, and clozapine are just a few examples, but the current literature is unfortunately inconsistent and reported data is contradictory, which is why we support additional studies in this context. Moreover, we further argue the utility of studying how distinct controlled substances influence microbial communities, considering that ketamine is proved to alleviate depressive-like behavior as opposed to amphetamine and phencyclidine, which are known substances to trigger SCZ-like symptoms in experimental models. Probiotics may be regarded as the most consequential vehicle through which the gut flora can be successfully influenced, in adequate doses exerting a beneficial role as an alternative approach to alleviate SCZ symptoms.

## 1. Introduction

SCZ is a multifaceted functional chronic and debilitating neuropsychiatric brain disorder that portrays a phenotype that incorporates manifestations from a dual spectrum of clinical signs, through which, differential diagnosis could be validated [1]. Thus, SCZ depicts five subtypes (Paranoid, Catatonic, Residual, Disorganized, and Undifferentiated) according to the fifth edition of the Diagnostic and Statistical Manual of Mental Disorders (DSM-5) [2].

While positive symptoms include psychosis-like hallucinations, delusions that are paranoid, and disorganized speech that surrounds conversations with or about the patient, negative ones revolve around anhedonia, the lack or absence of expressiveness, motivation, and social interactions, which promote withdrawal, psychomotor impairment such as catatonia, and gross posturing [3,4].

From the work of Kraepelin and Quen [5], until the reassessment of the negative symptoms as primary and positive symptoms as secondary nearly four decades ago [6,7,8,9], the distinction between dimensions is fundamental in understanding the functional limitations. This concept originated in the field of neurology and was later extrapolated into psychiatry, thus allowing a description of this disorder in terms of symptom domains.

The positive symptoms may be handled effectively through antipsychotics and are mostly related to psychiatric or medical comorbidities, adverse effects due to treatment, or environmental factors; little options exist for the core component of SCZ. On the other hand, the negative symptoms are intrinsically responsible for long-term morbidity and poor functional outcome persisting from DSM-III to DSM-5. This core dimension can be managed via careful examination, convenient identification, and the requirement of adequate therapy [10,11,12]. Currently, the actual prevalence is hard to establish since studies that rely on medical records, interviews, household surveys, and estimates in the US, oscillate between 0.25% and 0.64% [13,14,15], and between 0.33% and 0.75% on a global scale [16,17]. Hence, SCZ presumably affects around 24 million individuals worldwide, significantly impacting the resources of the healthcare systems [18].

The onset of SCZ varies in a sex-dependent manner; for example, late adolescence—the early twenties in males and early twenties-thirties in females [19,20]. These percentages reflect the predisposition toward suicide (4.9%) and other life-threatening conditions relative to the general population: usually two, or up to three times higher [21].

Despite the extensive research to decipher the background mechanisms of SCZ, the underlying interconnection is still insufficiently understood. Consequently, apart from the environmental hypothesis [22], it was uncovered that SCZ possesses a genetic substrate and it might be the result of variations of common, rare, copy number variants [23,24,25], and de novo mutations [26], except those already reported [27].

As a countermeasure to balance the conventional methods of choice [28], antipsychotics are the cornerstone of management strategies for patients diagnosed with SCZ. However, the medication dedicated in treating the symptoms amplify the predisposition toward various side effects [29].

Unfortunately, the role of gastrointestinal microflora (GM) has been neglected, as well as its involvement in shaping human development [30]. Recent studies have started to arise and suggested the beneficial outcomes obtained following the administration of derived supplements or specific techniques that revolve around the host’s eubiosis re-establishment [31].

Therefore, the present manuscript aims to gather together data regarding the use of pro-, pre- and synbiotics alongside fecal microbiota transplantation (FMT) and microbial transfer therapy (MTT) in SCZ, and how they may or not diminish the associated symptomatology and could be implemented in future clinical practice.

## 2. Methodology

The current narrative manuscript respects the guideline previously issued by Green et al. [32].

### 2.1. Database Searching Strategy

The relevant information until inception (November 2022) was founded on searching four scientific databases: PubMed/Medline, ISI Web of Knowledge, Scopus, and ScienceDirect.

The searching strategy consisted of a combination of keywords that contain “schizophrenia”, alongside “probiotics”, “prebiotics”, “synbiotics”, “fecal microbiota transplantation”, and “microbial transfer therapy”.

The following PubMed/Medline string was adopted: schizophrenia [Title/Abstract] AND probiotics [Title/Abstract] AND prebiotics [Title/Abstract] AND synbiotics [Title/Abstract] AND fecal microbiota transplantation [Title/Abstract] AND microbial transfer therapy [Title/Abstract].

We restricted the searches in PubMed/Medline to experiences performed in human patients. In ISI Web of Knowledge, Scopus, and ScienceDirect, we confined searches to research articles conducted in the pre-established interval of interest (2018–2022).

### 2.2. Inclusion Criteria

Eligible research were articles written in the English language and conducted in the predetermined interval (2018–2022) that included human patients, and excluded those performed on experimental models.

### 2.3. Exclusion Criteria

Articles written in another language than English, case reports or series, reviews or systematic reviews, meta-analyses, letters to Editors, editorials, opinions, responses, comments, conference posters or abstracts, work protocols, computational simulations, and preprints were not considered suitable.

### 2.4. Study Selection

All seven authors reviewed the information in the titles and abstracts of the retrieved results. Those considered eligible were screened based on the complete content, and all discrepancies were solved by consent with A.C. and R.D.

**Figure 1:**
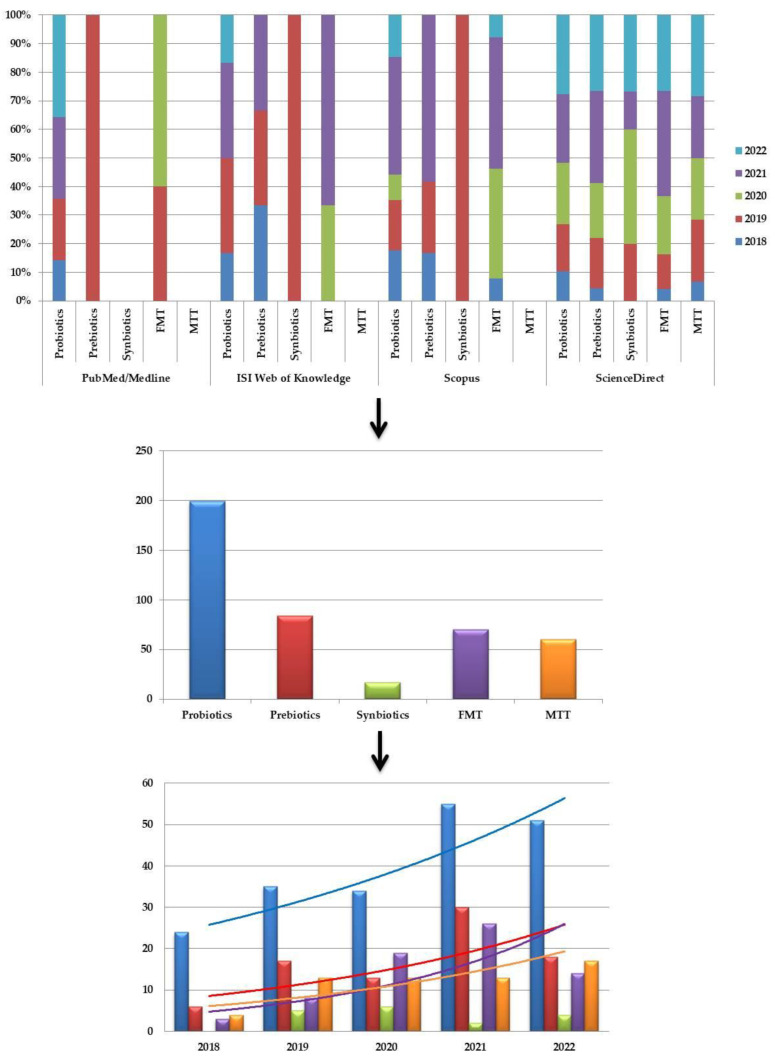
Diagram presenting the percentages of studies published per year depending on the therapeutic approach and the overall number, and the with the associated exponential trendline, except “synbiotics” due to the absence of results for the year 2018.

A total of *n* = 430 studies were returned from the predetermined interval (2018–2022), from which *n* = 37 (8.60%) were published in 2018, *n* = 78 (18.13%) in 2019, *n* = 85 (19.76%) in 2020, *n* = 126 (29.30%) in 2021, and *n* = 104 (24.18%) in 2022, if taken chronologically (Figure 1).

Based on the database explored, *n* = 20 studies were identified in PubMed/Medline, *n* = 13 in ISI Web of Knowledge, *n* = 60 in Scopus, and *n* = 337 in ScienceDirect. Depending on the combination of keywords applied, *n* = 199 studies were identified using “probiotics”, *n* = 84 for “prebiotics”, *n* = 17 for “synbiotics”, *n* = 70 for “fecal microbiota transplantation”, and *n* = 60 for “microbial transfer therapy” (Figure 1).

After manually removing duplicates, only *n* = 22 met the initial eligibility criteria and were subsequently considered for the inclusion in this manuscript. From that number, only *n* = 10 were included and summarized in Table 1, obtaining a total of *n* = 990 patients.

The authors sequenced the following: V3-V4 in *n* = 5 [33,34,35,36,37], V4 in *n* = 4 [38,39,40,41] and internal transcribed spacer (ITS) regions in *n* = 1; ITS1F; CTTGGTCATTTAGAGGAAGTAA, ITS2R’ GCTGCGTTCTTCATCGATGC [42]. Moreover, MiSeq was the most utilized instrument for sequencing in *n* = 6 [34,35,36,38,39,42] manuscripts, *n* = 2 for HiSeq 2500 [37,41], *n* = 1 for HiSeq 2000 [40], and *n* = 1 for NovaSeq 6000 [33].

**Table 1:** Summarizing overview of the main microbial alterations that occur in SCZ.

No. of Patients	Hypervariable Region Primers Sequencer	Microbial Ratio	Year of Publication	Reference
*n* = 117 53/64	V3-V4 341F 5′-GGACTACHVGGGTWTCTAAT-3′ 805R 5′-ACTCCTACGGGAGGCAGCAG-3′ HiSeq 2500	*Proteobacteria* ↑ *Firmicutes* ↓ *Succinivibrio* ↑ *Megasphaera* ↑ *Collinsella* ↑ *Clostridium* ↑ *Klebsiella* ↑ *Methanobrevibacter* ↑ *Blautia* ↓ *Coprococcus* ↓ *Roseburia* ↓	2018	[37]
*n* = 29 12/17	V3-V4 357F 5′-CGCTCTTCCGATCTCTGTACGGRAGGCAGCAG-3′ 806R 5′-CGCTCTTCCGATCTGACGGACTACHVGGGTWTCTAAT-3′ MiSeq	*Parabacteroides ↑*	2019	[34]
*n* = 37 21/16	V4 515F 5′GTGCCAGCMGCCGCGGTAA-3′ 806R 5′-TAATCTWTGGGVHCATCAGG-3′ MiSeq	*Alistipes* ↑ *Actinobacteria ↑*	2019	[39]
*n* = 50 25/25	V4 HiSeq 2000	*Proteobacteria ↓* *Anaerococcus ↑* *Haemophilus ↓* *Sutterella ↓* *Clostridium ↓*	2019	[40]
*n* = 194 40/85/69	V4 515F 5′GTGCCAGCMGCCGCGGTAA-3′ 806R 5′-TAATCTWTGGGVHCATCAGG-3′ MiSeq	*Christensenellaceae* ↑ *Enterobacteriaceae* ↑ *Pasteurellaceae* ↓ *Turicibacteraceae* ↓ *Escherichia* ↑	2020	[38]
*n* = 26 10/16	ITS1F CTTGGTCATTTAGAGGAAGTAA ITS2R GCTGCGTTCTTCATCGATGC 338F 5′- ACTCCTACGGGAGGCAGCAG-3′ 806R 5′-GGACTACHVGGGTWTCTAAT-3′ MiSeq	*Proteobacteria* ↑ *Faecalibacterium* ↓ *Lachnospiraceae* ↓ *Chaetomium* ↑ *Trichoderma* ↓	2020	[42]
*n* = 168 84/84	V4 515F 5′GTGCCAGCMGCCGCGGTAA-3′ 806R 5′-TAATCTWTGGGVHCATCAGG-3′ HiSeq 2500	*Actinobacteria* ↑ *Deltaproteobacteria* ↑ *Actinomycetales* ↑ *Sphingomonadales* ↑ *Rhodocyclales* ↓ *Sphingomonadaceae* ↑ *Alcaligenaceae* ↓ *Enterococcaceae* ↓ *Leuconostocaceae* ↓ *Rhodocyclaceae* ↓ *Rikenellaceae* ↓ *Eggerthella* ↑ *Megasphaera* ↑ *Enterococcus* ↓ *Akkermansia muciniphila* ↑ *Bifidobacterium adolescentis* ↑ *Clostridium perfringens* ↑ *Lactobacillus gasseri* ↑ *Megasphaera elsdeniis ↑*	2020	[41]
*n* = 118 32/29/29/28	V3-V4 MiSeq	*Firmicutes* ↓ *Bacteroidetes* ↑ *Bacteroidaceae* ↑ *Rikenellaceae* ↑ *Tannerellaceae* ↑ *Bacteroides thetaiotaomicron* ↑ *Bacteroides uniformis* ↑ *Parabacteroides goldsteinii ↑*	2022	[35]
*n* = 161 90/71	V3-V4 341F 5′-CCTACGGGNGGCWGCAG-3 ’ 785R 5′-ACTACHVGGGTATCTAATCC-3′ MiSeq	*Faecalibacterium* ↓ *Roseburia* ↓ *Actinomyces* ↑ *Butyricicoccus* ↓ *Prevotella* ↑	2022	[36]
*n* = 90 45/45	V3-V4 341F 5′-CCTACGGGNGGCWGCAG-3′ 805R 5′-GACTACHVGGGTATCTAATCC-3′ NovaSeq 6000	*Bacteroidetes* ↓ *Fusobacteria* ↓ *Firmicutes* ↑ *Verrucomicrobia* ↑ *Synergistetes* ↑ *Christensenella* ↑ *Desulfovibrio* ↑	2022	[33]

↑—increase; ↓—decrease.

## 3. Discussion

### 3.1. Unique and Joint Effects of the Composition of Gut Microflora

#### 3.1.1. Probiotics

A team of experts in the field have tried throughout the years to implement a regime based on *Bifidobacterium breve* A-1 (10^11^ colony-forming units (CFU)/day) for one month on two occasions and centralize the scores according to the Hospital Anxiety and Depression Scale (HADS) and Positive and Negative Syndrome Scale (PANSS). After four weeks of treatment, the authors noted an elevation in the severity of negative symptoms among responders and a reduction in the intake of dairy products with a relative abundance of *Parabacteroides*. Although different from the baseline, the expression of interleukin (IL)-22 and tumor necrosis factor (TNF)-related activation-induced cytokine (TRANCE) amplifies in responders, in contrast with non-responders [34]. Subsequently, Yamamura and collaborators [43] reproduced the pilot study of Okubo et al. [34]. They pinpoint the elevation of the lipid and energy metabolism in the so-called responders, compared with non-responders arguing the necessity of establishing the microbial profile genes at the baseline prior to the initiation of probiotic therapy to discriminate the possible trajectory pre- and post-intervention.

#### 3.1.2. Vitamin D

Perhaps the sole study existing in the current literature is the experience of Ghaderi et al. [44] where they tested, in a randomized, double-blind, placebo-controlled trial (IRCT2017072333551N2), the outcomes of supplementation with vitamin D (50,000 IU) along with LactoCare^®^ (8 × 10^9^ CFU/day)—*Lactobacillus reuteri*, *Lactobacillus fermentum*, *Lactobacillus acidophilus*, and *Bifidobacterium bifidum* (2 × 10^9^ each)—for twelve weeks. First, there was an improvement in the general (*p* = 0.004) and total scores of PANSS (*p* = 0.01), which enhanced the total antioxidant system (*p* = 0.007) by decreasing the malondialdehyde (MDA) (*p* = 0.01) level in the plasma, therefore preventing inflammation, and the high-sensitive (hs) C-reactive protein (CRP) level (*p* = 0.001) that finally crystallized under the status of the following: fasting plasma glucose (FPG) (*p* = 0.01), insulin concentrations (*p* < 0.001), homeostatic model assessment-estimated insulin resistance (HOMA-IR) (*p* < 0.001), triglycerides (*p* = 0.01), total cholesterol levels (*p* = 0.04), and the total and high-density lipoprotein (HDL)-cholesterol ratio (*p* = 0.04).

#### 3.1.3. Selenium

Another randomized, double-blind, placebo-controlled trial revolved around the benefits of LactoCare^®^ (8  ×  10^9^ CFU/day) containing *Lactobacillus acidophilus*, *Bifidobacterium lactis*, *Bifidobacterium bifidum*, and *Bifidobacterium longum* (2 × 10^9^ each) and selenium (200 μg/day) co-administration for twelve weeks in the study of Jamilian and Ghaderi [45] (IRCT20170513033941N41). Analogous to the previous team, results showed a significant improvement in the general PANSS scores (*p* = 0.03). Congruent with vitamin D, there was an increase in the total antioxidant capacity (TAC) (*p* = 0.002) and total glutathione (*p* = 0.008), coupled with a reduction of high-sensitivity hs-CRP levels (*p* = 0.001). As expected, in this manner, the co-administration highlighted a decrease in fasting glucose (*p* < 0.001), insulin (*p* = 0.002), HOMA-IR (*p* < 0.001), and in the quantitative insulin sensitivity check index (QUICKI) (*p* < 0.001) by comparison with placebo.

A brief summary of the joint effects of the above-mentioned supplements is presented in Table 2.

#### 3.1.4. Olanzapine

From this evidence, additional perspectives and interests towards the role of *Bifidobacterium*, *Lactobacillus* and *Enterococcus* (1 × 10^7^ CFU each [46]/ ≥ 5.0 × 10^7^ CFU/g [47]) in the amelioration of olanzapine-induced body weight arose [46,47]. Yang et al. [46] emphasized a between-group difference, after four weeks treatment, in weight (*p* < 0.05) and body mass index (BMI) (*p* < 0.05) that lasted for eight to twelve weeks of treatment (*p* > 0.05) with no differences in terms of appetite during the analyzed period (*p* > 0.05); a non-statistical significance mean time from olanzapine initiation to appetite changes was also obtained (*p* = 0.22). Moreover, two randomized controlled trials (RCTs) conducted by Huang et al. [47] have been conducted to evaluate the benefits brought by Bifico. In the first twelve-week study, no effects of the probiotics on weight gain (*p* < 0.005) were observed, whereas in the second study, probiotics and dietary fiber exerted a beneficial impact (*p* = 0.007) per insulin resistance index (IRI) value (*p* < 0.005/*p* < 0.001), particularly in olanzapine monotherapy, but not in olanzapine combined with probiotics and dietary fiber (*p* = 0.35). Another publication from the same team reported in a twelve-week intervention with dietary fibers, probiotics, both, or placebo, intervention with dietary fibers, and probiotics are superior to any other approach in reducing weight the BMI, and total cholesterol [35].

#### 3.1.5. Risperidone

Another publication by Yuan et al. [48] carried out on first-episode drug-naïve psychotic patients for twenty four weeks demonstrated significant gut and metabolic parameter changes. Risperidone led to an increase in body weight, BMI, fasting blood-glucose, triglycerides, LDL, hs-CRP, superoxide dismutase (SOD), and HOMA-IR (*p* < 0.001), and the depletion of the so-called beneficial bacteria, among which are *Bifidobacterium* spp. and *Lactobacillus* spp., and *Escherichia coli* (*p*s < 0.001) to the detriment of *Clostridium coccoides* (*p*s < 0.001). Risperidone treatment for twenty four weeks promoted an increase in the abundance of *Bifidobacterium* spp. and *Escherichia coli* (*p*s < 0.001), while a notable decrease was evident in *Lactobacillus* spp. And the *Clostridium coccoides* group (*p*s < 0.001); the hierarchical multiple linear regression analysis indicated solid correlations of the metabolic and gut microflora translocations with the relative abundance of *Bifidobacterium* spp. exclusively, body weight (*p* = 0.009) and BMI (*p* = 0.008).

#### 3.1.6. Amisulpride

In a pilot study, Zheng et al. [49] aimed to analyze the consequences of amisulpride (400–1200 mg/kg) treatment for four weeks by applying biochemical and molecular biology protocols. As observed in the case of all atypical antipsychotics (AAP), it caused an increase in the levels of the short-chain fatty acid (SCFAs)-producing bacteria *Dorea* and *Butyricicoccus* and of those that are potentially pathogenic, such as *Actinomyces* and *Porphyromonas* with persistence at a high ratio of *Desulfovibrio*. Moreover, the intervention led to a downregulation of butanoate and elevation of IL-4 compared with IL-6.

#### 3.1.7. Clozapine

Gorbovskaya et al. [50] designed a two-phase protocol, currently underway, regarding chronic clozapine (200/252.3 mg) treatment to deepen understanding of the underlying mechanisms of antipsychotic medications and metabolic abnormalities and possibly the transplantation of human samples.

#### 3.1.8. Multiple Antipsychotics

Flowers et al. [39] conducted a cross-section cohort study investigating how antipsychotics might disturb the host’s eubiosis and evaluate the translocations in a sex-dependent manner alongside prebiotic raw, unmodified potato starch for two weeks. As opposed to the non-APP users, those that followed an APP treatment had distinct signatures, particularly the females within the *Lachnospiraceae* family, and *Akkermansia* and *Sutterella* genera between the two investigated groups; there was also an increase in the *Actinobacteria* phylum in second-generation antipsychotic (SGA)-treated patients and with an increased fractional representation of *Alistipes* in non-APP users that showed resistant starch supplementation.

### 3.2. In-Depth Overview

With all the information, Li et al. [51] further offered novel data into plasma lipid metabolism through either standard enzymatic protocols using an automated analyzer or analytical measurements by gas chromatography-mass spectrometry (CG-MS), and cognitive performance based on the MATRICS Consensus Cognitive Battery (MCCB). Besides the high serum levels of SCFAs, and acetic acid, acetic acid/propionic acid ratio, and low cognitive scores (*p*’s < 0.05), there was also a positive correlation between the lipid levels and acetic acid/acetic acid ratio within the same group (*p*’s < 0.05); however, the low-density lipoprotein (LDL) × acetic acid/propionic acid ratio might be viewed as a predictor of the MCCB working memory.

Moreover, the abnormal levels of fecal amino acids may be a pointer towards the severity of the clinical signs exhibited, as Jansma et al. [52] suggest. In this context, they investigated the associations between fecal level metabolites using proton nuclear magnetic resonance (^1^H NMR), clinical parameters, and dietary components; they found a positive relationship between one non-essential and two essential amino acids, among which, alanine, leucine, and valine, as well as with dairy intake, were used as alternatives to improve the severity of symptoms.

Continuing with this concept, two teams had a common goal of deciphering how microorganisms disrupt the mucosal immune system. Xu et al. [41] showed an active function of glutamate synthase in affected patients, depicted by the immunoglobulin A (IgA) levels and of the bacterial translocation biomarker, including the lipopolysaccharide (LPS)-binding protein and the cluster of differentiation (CD)14. In the other circumstance, Ling et al. [36] proved in their observational study that neuroinflammation is a phenomenon characteristic of psychiatric disorders, as strengthened by the level of IL-1β, which was significantly upregulated and antithetical to that of interferon-gamma (IFN-γ), presumably because of the β-diversity following the linear discriminant analysis effect size (LEfSe).

Mechanistically speaking, one contributor that may exacerbate this pathology is the air pollution alongside long exposure to nitrogen dioxide (NO_2_). Yi et al. [53] discussed how particulate matter of NO_2_, carbonic oxide (CO), and ozone (O_3_), with a diameter of 10 µm (PM_10_) or are small (PM_2.5_), induce gut alterations from 2.68% to 10.77% (*p* < 0.05). Network analyses also indicate an association with the liver marker function, and with three phyla *Firmicutes*, *Actinobacteria*, and *Proteobacteria*.

Interestingly, Zhu et al. [54] performed a metagenome-wide association study (MWAS) aiming to explore the differences between microbial communities and found that medication-free patients harbor many facultative anaerobes entities such as *Lactobacillus fermentum*, *Enterococcus faecium*, *Alkaliphilus oremlandii*, *Cronobacter sakazakii*, and *Cronobacter turicensis*; this dysbacteriosis engages the SCFAs synthesis, tryptophan (Trp) metabolism, including higher kynurenic acid (KYNA) levels, and metabolisms, particularly glutamate and gamma-aminobutyric acid (GABA), serotonin (5-HT) and dopamine (DA).

One eloquent example of a gastrointestinal symptom that better depicts the interconnection between the brain and gut is hypomotility. The α-diversity increase Chao 1, ACE, and vitamin B6, according to the results of Xu et al. [33] in constipated patients. However, these arguments are contradicted by the observations of Ma et al. [38], as they noted an increase in the *Enterococcaceae* family levels. In the same study, significant changes in the volume of the right middle frontal gyrus (rMFG) following magnetic resonance imaging (MRI) appeared to be related to a specific composition of the microbiota. Among the most pregnant patients, inconsistencies were counted in the taxonomic structure, but not within the syntheses pathways as previously suggested.

Intriguingly, the current data is indeed inconsistent, since Nguyen and Shen et al. [37,40] argue that there are no differences among the analyzed samples, while Ma and Xu et al. [38,41] indicate a significant alteration in the α-diversity; rather, inter-group differences in unweighted Unifrac and Bray-Curtis distances were observed, with two reporting an increase in the abundance of the phylum *Proteobacteria* [37,38], two without notable differences [41,55], and one describing a decrease [40]. Finally, a small pilot study centered upon the differences in drug-naïve patients reflected a relative reduction in α-diversity, more specific SCFA-producing bacteria *Faecalibacterium* and *Lachnospiraceae,* with an increase in the abundance of the potentially harmful phylum *Proteobacteria* and *Romboutsia* [42].

### 3.3. Future Directions of Research

To our surprise, there is little evidence in the literature regarding the use of prebiotics, and none of synbiotics, possibly because of its inefficiency due to the current abnormal microbial ratio in SCZ. However, the ingestion of prebiotic formulation galacto-oligosaccharide (B-GOS^®^) proved to improve cognitive flexibility in rats [56], also improving the global cognitive performance per Brief Assessment of Cognition in Schizophrenia (BACS) in human patients [57] as opposed to placebo [58]. Regardless of this evidence, replication at a larger scale is mandatory, which could also include the assessment of positive and negative symptoms to understand the therapeutic potential of prebiotics. It appears that B-GOS^®^ may increase the responses of prefrontal cortex (PFC) pyramidal neurons, in parallel to elevating the hippocampal brain-derived neurotrophic factor (BDNF) levels [59], and the cortical expression of two distinct N-methyl-D-aspartate (NMDA) subunits, particularly GluN2B and GluN2A [56,59].

In addition, there are FMT and MTT dedicated to gut flora reconstruction that have created controversies due to the personalized nature of the microbial communities. Although FMT is preferred and known to be a safe and well-tolerated approach, there are numerous ethical and legal provisions combined with the absence of stool banks and working protocols among human patients made to standardize the working protocol. Moreover, multiple limitations must be overcome, including finding eligible donors, the aesthetics of the procedure, and the elimination risk of cases of adverse effects. Zhu et al. [60] transferred fecal microbiota from drug-free SCZ patients into pathogen-free models. Abnormal phenotypic peculiarities, such as psychomotor hyperactivity, alongside impaired memory and learning capacity, were noted. At a molecular level, FMT caused an elevation of the kynurenine–kynurenic acid (KYNA) pathway of tryptophan (TRP) in the brain and periphery, and of basal extracellular dopamine (DA) in PFC and 5-hydroxytryptamine (5-HT) in the hippocampus, in contrast with the mice that received feces from the healthy individuals. Within the same study, the authors noted an increase of both KYNA synthesis and kynurenine aminotransferase II (KAT II) activity in cultured hepatocytes and forebrain cortical slices following colonic luminal filtrates that culminated in sixty donor-derived specie identification between the groups.

One promising and yet underexplored field of interest, that is still in its infancy, may be illustrated by the action of various agents that usually trigger an SCZ-related phenotype and modifications at the gut flora level. Although no conclusive evidence exists in the literature, it has started to gain interest in the antidepressant-like potential of certain candidate drugs. Fortunately, several have been already tested on murine models and humans; however, future research is mandatory to fully understand the mechanisms behind SCZ and the interplay with gut flora.

#### 3.3.1. Ketamine

(R, S)-ketamine may restore, to some extent, gut disturbances in chronic social defeat stress (CSDS) mice, being characterized by a decrease of *Tenericutes* and an increase of *Actinobacteria,* as indicated by Qu, and Yang et al. [61,62]. Both ketamine enantiomers promote a significant amelioration of *Deltaproteobacteria*, whereas (R)-ketamine proved to be more potent by normalizing and attenuating the reduction and the overall ratio of *Bacteroidales*, *Clostridiales*, *Ruminococcaceae*, *Mollicutes*, and *Butyricimonas* than (S)-ketamine or lanicemine. An inflammatory mice model of LPS-induced depressive-like behavior has been generated recently by Huang et al. [63]. *Coriobacteriia* might actually be responsible for this phenomenon, i.e., for the high immobility tendency in the forced swimming test (FST) effect that is countered by *Actinobacteria*: microorganisms believed to be markers of the associated anti-depressive potential of ketamine. However, those evaluating the effects of low-dose ketamine administration on rats found a striking amplification of *Turicibacter* related to ketamine by 26-fold, also of *Lactobacillus* by 3.3-fold, and *Sarcina* by 42-fold, and a restriction in the development of opportunistic pathogens such as *Mucispirillum* and *Ruminococcus,* according to Getachew et al. [64].

#### 3.3.2. Amphetamine

In addition, research from Yang et al. [65] performed on individuals with methamphetamine (METH)-use disorders reveal that they have a specific microbial composition and dysbiosis correlated with METH-induced conditioned place preference (CPP). The samples analyzed provided information about elevated concentrations of *Bacteroides* and *Faecalibacterium*, suggesting that the gut could be a modulator for METH-induced behavior and vulnerability in persons with psychotic syndromes.

#### 3.3.3. Phencyclidine

Nevertheless, Jørgensen et al. [66] treated murine models with sub-chronic phencyclidine (PCP) to model endophenotypes of SCZ and discovered slightly important alterations in the core microflora. These modifications were associated with poor object recognition memory performance and promoted a tendency of hyperlocomotion. Ampicillin abolished the increased locomotion, linked to an elevation in *Lachnospiraceae* and *Clostridiaceae* and an increase in *Roseburia*, *Clostridium*, and *Odoribacter*.

## 4. Conclusions

Based on the aspects discussed throughout this manuscript, it can be concluded that the (co-)administration of probiotics, particularly strains of *Lactobacillus* and *Bifidobacterium*, along with vitamin D and selenium, enhance the antioxidant system, preventing the inflammation, body weight related to olanzapine, and a plethora of other variables suggestive for metabolic dysfunctions. On the other hand, there are antipsychotics such as risperidone, amisulpride, and clozapine that have an opposing effect upon the host’s overall condition. Analyses performed clearly reveal systematic repercussions, reflected by the up- and downregulation of biomarkers upon a multitude of synthesis pathways and the immune system; however, there are also reports that raised contradicting evidence regarding the gut flora structure and taxonomic composition between the groups. Having that in mind, further studies are mandatory from our point of view to discriminate those factors that establish a cause–effect relation and how microorganisms that reside within our gut may alleviate or exacerbate the phenotype. Further, this is why we consider it important to assess the pivotal role of gut microflora in the cases of putative drugs, for example, ketamine, whose activity was demonstrated in experimental models to alleviate depression-like behavior. However, there are other controlled substances, including amphetamine and phencyclidine, still with an obscure trajectory until the associated disturbances of the gut microflora. To our surprise, we could not identify studies centered on the supposed therapeutic role of prebiotics, synbiotics, FMT, or MTT to date.

## Figures and Tables

**Table 2:** Observed commune benefic role following 50,000 IU vitamin/200 μg/day selenium alongside LactoCare^®^ in SCZ patients.

Vitamin D and Selenium	Reference
- Improvement in the general PANSS scores - Increase of the TAC - Reduction of the hs-CRP - Decrease of the FPG, insulin concentrations, and HOMA-IR	[44,45]

## Data Availability

The datasets used and analyzed during the current study are available from the corresponding author on reasonable request.
